# Advancing health equity: Why guideline development must prioritize fairness and justice

**DOI:** 10.1002/gin2.70015

**Published:** 2025-01-28

**Authors:** Omar Dewidar, Jordi Pardo Pardo, Juan Pablo Peña‐Rosas, Rebecca Thomas, Vivian Welch, Peter Tugwell

**Affiliations:** ^1^ Temerty Faculty of Medicine University of Toronto Toronto Ontario Canada; ^2^ Bruyere Research Institute University of Ottawa Ottawa Ontario Canada; ^3^ Ottawa, Hospital Research Institute University of Ottawa Ottawa Ontario Canada; ^4^ Global Health Initiatives, Department of Nutrition and Food Safety, World Health Organization Geneva Switzerland; ^5^ University of Liverpool Liverpool UK; ^6^ Faculty of Medicine University of Ottawa Ottawa Ontario Canada

**Keywords:** evidence‐based medicine, guideline, health equity, policy, public health, systematic reviews

## Abstract

Health equity should be regarded as a fundamental principle and a priority for all guideline development organizations. Yet, this principle has not been consistently prioritized in the creation of mainstream guidelines. In this commentary, we examine a real‐world example from leprosy management, where the initial lack of integration of health equity considerations in the guideline recommendations did not consider the potential impact of the recommendations on the health of populations most affected by leprosy. We also highlight subsequent changes in the guideline development process that reflect stronger consideration of health equity, addressing some of the previous issues propagated with historical practices. We also draw on other examples from several fields to further illustrate the impact of integrating health equity considerations in guidelines. Building on evaluations of guidelines for health equity and real‐world experiences, we highlight some of the common challenges in integrating health equity considerations in the guideline development process. We propose potential solutions using existing tools and frameworks and outlining key research priorities to further advance this goal.

## Key points

*Key Findings*

- Integrating equity considerations in guidelines can improve health outcomes, particularly for populations experiencing inequities, as demonstrated in examples such as leprosy management and tuberculosis.
- Challenges to integrating health equity in guidelines include lack of evidence, methodological complexities, resource constraints, and inadequate engagement of individuals with lived experiences of inequities.

*What this adds to what was known?*

- We highlight practical examples from published guidelines on how considering health equity in the development of guidelines has influenced recommendations and may have contributed to better health outcomes for populations experiencing inequities.

*What is the implication and what should change now?*

- Scalable methods for integrating health equity considerations into guideline development are needed to accommodate variations in resource availability and the evolving evidence‐base.
- The research community is encouraged to address the unsolved methodological challenges for integrating health equity in guidelines such as accounting for variations in context and addressing intersectionality.

## 1 ADDRESSING HEALTH EQUITY SHOULD BE A PRIORITY IN GUIDELINES

Health equity is a primary concern in the political and policy realms for the World Health Organization (WHO) and numerous global entities. It is integral to fulfilling their overarching objective of all peoples to attain the highest possible level of health.^1^ Health equity is defined as the absence of unfair, avoidable, or remediable differences among population groups, whether these groups are defined socially, economically, demographically, or geographically or by other dimensions of inequality (e.g., sex, gender, ethnicity, disability, or sexual orientation).^2^ Addressing the social determinants of health is widely recognized as the primary approach to achieving health equity. Practice guidelines can serve as powerful tools for addressing these determinants by tailoring recommendations for populations experiencing inequities or integrating health equity considerations within broader strategies. In turn, these guidelines would address the distinct needs of populations experiencing inequities and foster more equitable health outcomes.^3^

As a leading global public health entity, the WHO has taken multiple initiatives to improve equity in their guideline development processes. This includes dedicating a chapter on equity, human rights, gender, and social determinants in the WHO guideline development handbook,^4^ adopting the GRADE approach and Evidence to Decision framework,^5^ which incorporates equity as a criterion, and developing the WHO‐INTEGRATE^6^ framework which is suited to develop system level recommendations. More recently, the WHO have promoted gender equality and human rights in their research practices through adopting the SAGER^7^ and GATHER guidelines,^8^ exemplifying their commitment through mandating sex‐specific data and outcome reporting.

Health equity should be a priority for all guideline‐producing organizations, not just those published by the WHO. It is a shared responsibility among all interest‐holders^9^ in global health, including policymakers, researchers, practitioners, and patients and members of the public. While addressing health equity in guidelines is a critical step towards promoting fairness and justice in health outcomes across diverse populations, the actual impact on reducing health inequities will depend on several factors such as the redistribution of health benefits across diverse populations,^10^ management of opportunity costs associated with addressing health inequities^11^ and the implementation and uptake of the guidelines.^12^ Some interventions that are intended to reduce health inequities in certain populations could simultaneously create inequities in other populations.^13^, ^14^ Therefore, it is important to not consider the impact of health equity in isolation, instead consider other critical factors such as cost‐effectiveness^15^, ^16^ when developing the guideline recommendations. Continuous evaluation of these factors is also required to ensure that health equity considerations are achieving their intended outcomes.

Health equity should be considered as a normative principle in the development of all guidelines. However, we acknowledge that the extent to which equity considerations are integrated in guidelines should depend on the context and the population addressed by the guideline. This could be guided by how sensitive the guideline is to health equity.^3^ Furthermore, efforts should be made to ensure that health equity considerations are evidence‐based,^17^ that is, empirical evidence and or lived experiences. In this manuscript, we illustrate the importance of integrating health equity considerations into guideline development by using examples from WHO guidelines that have successfully incorporated health equity considerations and describing their impacts. Additionally, we discuss common barriers to developing evidence‐based health equity considerations and propose solutions to overcoming them, as well as areas for future research.

## 2 RISK OF NOT INCLUDING HEALTH EQUITY CONSIDERATIONS IN GUIDELINES

Health equity is yet to be centered in the development of most guidelines.^18^, ^19^, ^20^ Centering health equity in the guideline means prioritizing and integrating considerations of fairness, inclusiveness, and justice throughout the development, implementation, and evaluation processes and products of the guideline.^21^

Despite several guidelines addressing topics, which are intertwined with increased stigma, human rights violations, and health disparities between groups, such as infectious diseases, there are notable gaps in health equity considerations in the guidelines. Recommendations that predominantly focus on populations experiencing inequities often lack rigorous evidence to support them,^18^, ^19^ fail to consider important intersecting causes of inequities, and do not address underlying barriers that key populations face when trying to access interventions.^20^

The historical management of leprosy provides a powerful example of the negative consequences of failing to consider health equity in guidelines. Early legislations focused on involuntary isolation of people with leprosy as an act to control the spread of the disease. These legislations were made without considering principles of health equity and human rights,^22^ which led to poorer health outcomes for leprosy patients, increased stigma of the disease, and human rights violations.^23^ The implementation of involuntary isolation policies led to the creation of leprosy colonies, where patients were socially isolated, sometimes for the rest of their lives.^23^, ^24^ The stigmatization associated with leprosy hindered early diagnosis of infected individuals as individuals with symptoms were often afraid to seek help due to the fear of isolation and did not seek treatment.^25^

Although leprosy was declared eliminated in most countries by 2010, it continues to affect several low‐ and middle‐income countries.^26^ Today's guidelines for managing leprosy have shifted dramatically from past practices, now emphasizing equity, community integration, and respect for human rights.^27^ These guidelines aim to reduce stigma, ensure early diagnosis and treatment, and promote the social inclusion of affected individuals. They advocate for community‐based care integrated into general healthcare services to increase accessibility and reduce stigma. Additionally, they recommend combating discrimination through education and awareness efforts.^28^

In 2021, leprosy is still an ongoing issue with Brazil, India and Indonesia accounting for 74.5% of the new leprosy cases worldwide.^29^ A recent report from the WHO^30^ highlights ongoing challenges, including limited access to quality healthcare services, low public awareness, and stigma that keeps individuals from seeking help early. However, current guidelines recommend community‐based care that integrates leprosy services into general healthcare, aiming to reduce stigma and support early diagnosis and effective treatment. They also focus on combating the historical stigma through education and awareness campaigns. International initiatives, such as the WHO Global Leprosy Strategy (2021–2030),^30^ specifically focus on reducing discrimination and improving access to care for marginalized populations. Nevertheless, challenges remain particularly in regions where deep‐rooted social and economic inequities exist, illustrating the importance of health equity‐centered guideline development that addresses both healthcare delivery and the broader social determinants of health.

## 3 IMPACT OF INTEGRATING HEALTH EQUITY CONSIDERATIONS IN GUIDELINES

Integrating equity in guidelines could be utilized to help achieve the global objectives set by the United Nations,^31^ specifically the Sustainable Development Goals (SDGs) 3 (Good Health and Well‐being) and SDG 10 (Reduced Inequalities). An example of this can be found in the WHO guideline on non‐surgical management of chronic primary low back pain (CPLBP) in adults, within primary and community care settings. The guideline describes health equity as one of its four guiding principles for its developement.^32^ The rationale behind this focus is because CPLBP is a leading cause of global disability across all age groups and genders, with a higher prevalence and disability rates among older individuals. Consequently, the guideline development group (GDG) developed 24 recommendations and one good practice statement, considering all adults, including older adults. The good practice statement advocates for providing quality, affordable mobility assistive products to adults, especially older individuals, based on personalized assessments. It also recommends addressing infrastructure barriers, such as installing ramps, to enhance accessibility.

Integrating health equity in guidelines also ensures that interventions reach those who need them the most, reducing the burden of disease among those most affected. The WHO consolidated guidelines on tuberculosis (Module 4: treatment of drug‐susceptible TB) exemplify this by focusing on key populations likely to experience inequities.^33^ These populations are defined as those with increased exposure to TB bacilli, limited access to health services, or heightened TB risk due to compromised immune function. Through the Stop TB Partnership, the WHO developed key population briefs emphasizing the inclusion of these populations in the development of both TB prevention and treatment guidelines.^34^ This inclusive approach aimed to improve treatment outcomes for populations experiencing inequities, ultimately reducing economic burdens such as lost productivity and increased healthcare costs.^35^

Another example is shown in the WHO guideline on syphilis screening for pregnant women which addresses economic burdens in low‐income countries by suggesting alternative cost‐effective screening and treatment strategies.^36^ These strategies are designed to be feasible within the constraints of limited healthcare resources and infrastructure often found in these regions. By recommending scalable and adaptable approaches, such as point‐of‐care testing or simplified treatment regimens, the guidelines help to reduce the financial strain on healthcare systems and individuals. This, in turn, promotes greater accessibility to essential maternal health services, ensuring that even economically disadvantaged populations can receive timely and effective care for syphilis during pregnancy.

## 4 HOW TO ADDRESS THE LACK OF HEALTH EQUITY CONSIDERATIONS IN GUIDELINES

### 4.1 Perceived methodological barriers

Although many guideline development organizations recognize the importance of integrating health equity in guidelines, several barriers hinder this process.

One common barrier is the lack of health equity evidence from primary studies,^37^, ^38^, ^39^, ^40^ making it difficult to incorporate evidence‐based health equity considerations into guidelines. Additionally, GDGs often face constraints in time, funding, and personnel which impedes the integration of health equity considerations as they tend to requires additional resources.^41^ As a result, there is a perceived inability to thoroughly address health inequities which inhibits some organizations from attempting to address them at all. Another barrier is the lack of connection of the guidance with individuals with lived experiences of inequities.^42^, ^43^ Implementation of several guidelines has indicated that the guidance produced does not align with the values and experiences of populations experiencing inequities.^44^ This list of barriers does not aim to be exhaustive but intends to highlight some of the common issues that guideline developers experience.

### 4.2 Solutions to overcome methodological barriers

#### 4.2.1 Lack of health equity evidence from primary studies

Efforts to improve clinical trial inclusivity align with improving the evidence base to inform health equity considerations in guidelines. For instance, the SENSITISE project aims to ensure that trials are designed and conducted to include diverse and historically underrepresented populations through targeted training and education.^45^ Similarly, the INCLUDE initiative, developed by the UK's National Institute for Health Research (NIHR) and Trial Forge,^46^ focuses on improving the diversity and inclusivity of participants in trials by identifying and addressing barriers that prevent certain groups from participating.

As part of Trial Forge's initiative, they created the INCLUDE Ethnicity Framework,^47^ which helps trialists determine which ethnic groups should be included in the tiral to ensure the results are generalizable and identify challenges in achieving this inclusivity. Additionally, the PRO‐EDI framework^48^ provides a structured approach to identify, measure, and address disparities in trial participation. In Australia, focused group discussions were conducted to explore ways to enhance ethnic diversity in trials.^49^ These discussions led to recommendations such as partnering with community leaders, translating materials, minimizing participant burden, and building trust to enhance inclusivity and representation in clinical research.^49^

Complete reporting is closely tied to the conduct of research, and both are essential for the integration of health equity considerations in systematic reviews and guideline recommendations. To address this need, health equity reporting guidelines have been developed, including the extension of the Consolidated Standards of Reporting Trials (CONSORT),^50^ the CONSolIDated critERtia for strengthening the reporting of health research involving Indigenous Peoples (CONSIDER) statement^51^ and the ongoing health equity extension of the Strengthening the Reporting of Observational Studies in Epidemiology (STROBE) guidelines.^52^ Adherence to these guidelines can help researchers ensure that health equity‐relevant evidence is adequately reported, facilitating the identification and analysis of disparities and helping guideline developers make informed health equity decisions.

‘Black swan events’ are unexpected events that have a high impact and that, after occurring, are rationalized in retrospect as if they had been predictable. This concept was popularized by philosopher and former banker Nassim Nicholas Taleb in his book ‘The Black Swan’.^53^ In the context of evidence‐informed guidelines and public health, black swan events can include pandemics, natural disasters, economic crises, or any unforeseen situation that dramatically alters health and healthcare patterns. Since these events are unpredictable, the available evidence may not be sufficient to guide decision‐making in new and complex situations or contexts. The evidence that informing the decision is based on accumulated evidence from previous studies. In unexpected situations, there may be no prior data that is directly applicable, forcing healthcare workers to adapt quickly and make decisions based on the best information available at the time. Even when the primary studies failed to report critical information around health equity, guidelines can reflect on the limitations of the evidence base to inform future research. By highlighting the current shortcomings, guidelines can inform future research by pointing at gaps on health equity data, prioritizing the needs of groups where addressing the uncertainty could make a greater difference.

The occurrence of a black swan event may require continuous re‐evaluation of health interventions and policies, since what worked in a normal context may not be effective in extraordinary circumstances. In such cases, priority should be given to lived experience as a minimum. In addition, health professionals could apply their clinical judgment and be flexible in their approach, using existing evidence as a guide, but also considering clinical experience and current circumstances.

#### 4.2.2 Constraints in time, funding, and personnel

To inform health equity judgments at the recommendation level, guideline developers need to seek evidence with a health equity lens. However, not all guideline organizations are equipped to do so due to the additional resources (time and personnel) required for these reviews. Dewidar et al. propose a scalable approach to help guideline developers identify equity‐relevant evidence.^54^ This approach has been applied in the context of chronic cannabis guidelines and is currently being piloted in other topics (Long‐COVID & extracorporeal membrane resuscitation).^55^

Depending on the available resources, guideline developers can implement additional approaches throughout the process to obtain evidence that informs health equity considerations. These approaches may include conducting health equity‐focused reviews or surveying individuals with lived experiences of inequities. Conducting these methods can help guideline developers better address health equity in their recommendations.

The core approach involves examining the identified evidence base for differences in baseline risk or baseline prevalence of outcomes, differences in effectiveness between populations, representation of populations experiencing inequities, and implementation considerations for these populations to ensure successful implementation. This method can be achieved with minimal additional financial resources and time by synergizing with the existing guideline process.

#### 4.2.3 Guidance lacks connection with individuals with lived experiences of inequities

Engaging individuals with lived experiences of inequities through consultation or shared decision‐making can provide valuable insights into the needs and barriers they face, making the guidance more relevant and effective. The MuSE consortium has developed guidance for interest‐holder engagement (in review),^56^ but there is still more work to be done to ensure equitable engagement.

Guideline panels should include individuals with lived experiences of inequities related to the guideline condition within the GDG. This includes patients, caregivers, clinicians, and other healthcare partners involved in patient care. Recall the chronic pain low back pain (CPLBP) guidelines. They incorporating members with lived experiences and expertise in gender, equity, and human rights in the guideline panel.^32^ These members were key in driving discussions and making judgments regarding health equity. It is important to note that as the scope of the guidelines broadens, the more likely health inequities would span a variety of factors (e.g., geography, protected characteristics, inclusion health groups, and socioeconomic status) that would make achieving a representative view very challenging. In such cases, a combination of public consultations and purposeful outreach could help collect all these diverse perspectives.

## 5 UNSOLVED BARRIERS FOR INTEGRATING HEALTH EQUITY CONSIDERATIONS IN GUIDELINES

Equity considerations can have global relevance or vary significantly depending on context and region. This has been illustrated in the case of leprosy.^27^ For instance, Colombian, Ghanaian, and Nepalese populations highlighted insufficient counseling on treatment duration and potential side‐effects like skin discoloration from clofazimine, as barriers for treatment uptake and continuation.^27^ Meanwhile, Indian populations identified stigmatization and unsupportive attitudes from healthcare providers as significant barriers, particularly in relation to preventive treatment (chemoprophylaxis) and screening of leprosy patients' contacts. In contrast, Ghanaian and Nepalese communities reported fewer concerns regarding stigma. This illustrates how context play a role in how populations experience inequities. Thus, guideline developers may struggle to factor in all these differences, especially when developing global or national guidance.

Despite our understanding of social determinants of health as the root cause of many inequities, and the availability of tools such as WHO‐INTEGRATE^6^ that aim to address system‐level issues, we still struggle to contextualize and understand these determinants sufficiently to provide practical solutions that meet the needs of populations experiencing inequities. Intersectionality is a critical framework for understanding how various forms of social stratification, such as race, gender, and other identity axes, intersect to create unique dynamics and effects, particularly regarding power and privilege.^57^ This approach highlights that individuals' experiences, and the causes of inequities are shaped by multiple, overlapping identities and social positions. It can be used to better conceptualize the causes of inequities and identify priority populations.^58^ In addition, planetary health equity—the intersection of environmental sustainability and health equity—further exacerbates these experiences of inequities.^59^, ^60^, ^61^ An illustrative example of the need for an intersectional approach can be seen in the WHO's mass drug administration (MDA) programs for lymphatic filariasis.^62^ Despite the recommendation, they have not yet achieved their target coverage in all areas, often leaving populations experiencing inequities behind. A qualitative research project found that intersecting determinants of inequity create power dynamics that inhibit equitable coverage of prevention programs, resulting in important sections of the population being missed.^63^ The researchers advocate prioritizing the ‘worst‐off’ to meet targets for NTD elimination. However, this intersectional lens has yet to be frequently adopted in WHO or other guidelines and is currently one of the most limited areas in terms of methodological guidance on how to address it.

An important limitation to addressing health equity considerations that guideline developers and their teams may lack the necessary preparation to understand health equity and address them confidently. This may be due to lack of awareness regarding health equity issues in the topic or the populations affected, and insufficient knowledge and expertise in applying health equity methods. Developing standardized training modules to address these barriers would be helpful to the guideline community and enhance the integration of health equity considerations in guidelines.

## 6 CONCLUSION

Health equity should be considered as a normative principle in the guideline development process, ensuring that it is not only acknowledged but embedded as a foundational element in the development of guidelines. However, several challenges impede the integration of health equity in guidelines, including the lack of health equity evidence, limited resources, and misalignment with the values and preferences of populations experiencing inequities. Solutions include considering health equity in primary studies by utilizing health equity reporting guidelines can help ensure that primary research adequately addresses health equity, developing scalable approaches for incorporating health equity considerations into guideline development processes, and engaging individuals with lived experiences of inequities in the guideline development process. These strategies can help ensure that health equity is centered in guidelines, ultimately leading to more inclusive and effective health interventions.

## AUTHOR CONTRIBUTIONS

Omar Dewidar, Jordi Pardo Pardo conceptualized the manuscript. Omar Dewidar prepared the first draft. Juan Pablo Peña‐Rosas and Rebecca contributed to preparing the first draft. All the authors provided critically reviewed the manuscript, provided edits and approved the submission of the manuscript.

## CONFLICT OF INTEREST STATEMENT

Omar Dewidar, Peter Tugwell, Jordi Pardo Pardo and Vivian Welch are members of the GRADE‐Equity Working Group and the Cochrane and Campbell Equity Thematic Group and have led several papers cited in this manuscript regarding equity in guidelines. Juan Pablo Peña‐Rosas is employed by the World Health Organization. The ideas expressed in this manuscript are the authors' own and do not necessarily reflect the decisions and policies of the WHO.

## ETHICS STATEMENT

The following word did not require patient consent, clinical trial registration, or research ethics approval.

## Data Availability

Data sharing not applicable to this article as no datasets were generated or analysed during the current study.
